# A Novel Randomized Trial Protocol for Evaluating Wound Healing Interventions

**DOI:** 10.1089/wound.2023.0058

**Published:** 2023-10-19

**Authors:** Richard Hillson Bull, Donna Clements, Agnes Juguilon Collarte, Keith Gordon Harding

**Affiliations:** ^1^ Accelerate CIC, Centenary Wing, St Joseph's Hospice, London, United Kingdom.; ^2^ CRN Eastern, Norfolk Community Health and Care Trust, Norwich, United Kingdom.; ^3^ North West Division (Central London, Hammersmith and Fulham and West London), St Charles Centre for Health and Wellbeing, London, United Kingdom.; ^4^ WWII Ltd (Welsh Wound Innovation Initiative), Welsh Wound Innovation Centre, Pontyclun, United Kingdom.

**Keywords:** venous leg ulcers, study design, self-controlled, neuromuscular electrostimulation

## Abstract

**Background:**

Randomized controlled trials using complete healing as an endpoint suffer from poor statistical power, owing to the heterogeneity of wounds and their healing trajectories. The Food and Drug Administration (FDA) has recently consulted with expert groups to consider percentage area reduction (PAR) of the wound over a 4-week period as a valid intermediate endpoint, creating the opportunity for more powerful study designs.

**Methods:**

A within-subject controlled study design comparing the PAR of venous leg ulcers (VLU) in patients over 4 weeks receiving different interventions. Twenty-nine patients received multilayer compression over 4 weeks, followed by neuromuscular electrostimulation (NMES) of the leg muscle pump in addition to compression for a further 4 weeks. Paired comparison was then made of PAR between the two phases. A second cohort of 22 patients received only multilayer compression throughout both 4-week phases.

**Results:**

Patients randomized to NMES saw a significant increase in healing rate compared with compression alone, whereas patients receiving compression only saw no significant change in healing rate throughout the course of the study.

**Conclusions:**

Intermittent NMES of the common peroneal nerve significantly accelerates the healing of VLU. It is well tolerated by patients and deserves serious consideration as an adjuvant to compression therapy. PAR is a useful metric for comparing the performance of wound healing interventions, and the self-controlled trial design allows sensitive discrimination with a relatively small number of subjects over a reasonably short trial period. The study is reported according to the CONSORT reporting guidelines.

**Clinical Trial Registration:**

NCT03396731 (ClinicalTrials.gov).

**Figure:**
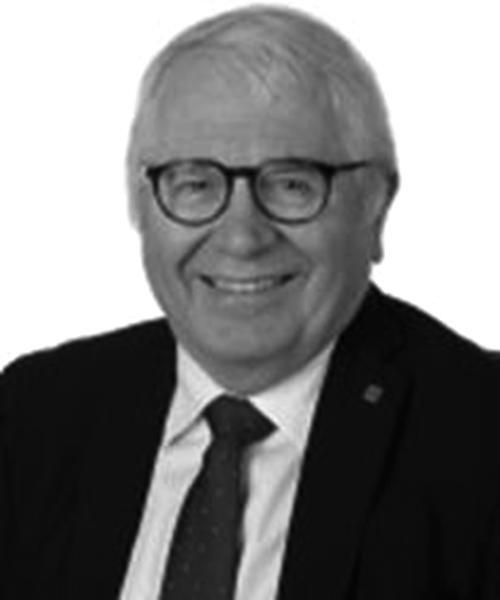
Keith Gordon Harding, FRCS

## INTRODUCTION

The management of venous leg ulcers (VLU) in the United States alone costs >$14 billion annually,^1^ and represents a significant burden worldwide, both financially and socially.^2^

A systematic review^3^ considered the effectiveness of a long list of interventions currently indicated for healing VLU, including: compression bandages and stockings, topical negative pressure, oral pentoxifylline, laser treatment, skin grafting, superficial vein surgery (perforator ligation, saphenous vein stripping), therapeutic ultrasound, leg ulcer clinics, leg elevation, and activity advice. Of these, only compression and pentoxifylline had statistically significant evidence to support their use.

The design of studies to evaluate wound healing interventions tends to follow the standard of evidence conventionally set by regulatory bodies and payors: a randomized controlled trial (RCT) consisting of intercohort comparison, with complete healing as the primary outcome.^4^ However, this trial design may not be the most appropriate for wound healing studies in the real world^5^ and may in part explain the scarcity of quality evidence. The great heterogeneity of ulcers (and their healing trajectories) makes it difficult to match control and intervention groups and necessitates many months of follow-up.^6^ Even if the intervention being evaluated has a very substantial effect, a very lengthy trial with a prohibitively large number (beyond the resources of the investigator to achieve) of stratified subjects would be required to observe that effect with any validity or statistical significance. Furthermore, it has been reported that the rate and frequency of ulcer healing is grossly overestimated in the literature^7^ causing many studies to be further under-powered and under-resourced at the outset.

There has been a call for alternate endpoints such as rate of wound closure over a specified period to be used for evaluations.^8,9^ This approach is advantageous not only because of the logistic advantage of a smaller window of observation (reducing cost and subject attrition while improving immediacy of results), but because it allows for more statistically powerful study designs, such as the self-controlled or within-patient controlled model that is deployed with success in other fields.^10^ Here, each subject's own rate of healing during a run-in phase is compared with that of the subject's own rate of healing during the treatment phase. This eliminates much of the heterogeneity and many of the confounders, thereby greatly improving the statistical sensitivity and power of the study. Within-subject control is not possible for a design using complete healing as a metric, as the healed wound is thereafter unavailable for comparison with the other intervention.

The Food and Drug Administration (FDA) has recently begun to review its position on acceptable study endpoints for wound studies.^11,12^ Whereas complete wound closure has hitherto been the only recognized endpoint, recent consultation with expert groups has led to the consideration of percentage area reduction (PAR) of the wound over a 4-week period as an endpoint.^13,14^ It has been demonstrated that PAR follows a near-linear trajectory over this period,^15^ and this makes it possible to observe any change in the rate of area reduction attributable to the introduction of a new intervention.

A recent review^16^ identified new technological developments for wound healing deemed worthy of further investigation, including activation of the leg muscle pumps by neuromuscular electrostimulation (NMES). Subsequently, a within-patient controlled study was conducted to evaluate the effects of a new intervention on the healing rate of venous leg ulcers.^17^ The study compared the wound margin advance (WMA) and PAR for VLU receiving 12 h per day intermittent NMES of the common peroneal nerve as an adjuvant to compression, compared with compression alone. The research protocol was approved by London—Riverside Research Ethics Committee (initial favorable opinion January 30, 2018), and all participants gave written informed consent. The primary endpoint in the study protocol was WMA, whereas PAR was calculated *post hoc*. Both metrics showed a substantial and significant effect for the intervention.

This article discusses the usefulness of the within-patient controlled study model (with particular reference to the metric of PAR over 4 weeks now being considered as a trial endpoint by FDA) and compares it with the relative insensitivity of intergroup control in the more conventional RCT model.

## METHODS

Sixty subjects, from 23 wound care clinics, with VLUs were randomized to two groups: one to receive standard of care (SOC) consisting of multilayer compression bandaging, and the other to receive NMES for 12 h per day in addition to SOC. Randomization allocation ratio was 1:1 using the Castor EDC platform with variable block sizes of 3 and 6.

NMES consisted of the geko™ device (Firstkind Ltd, Daresbury, United Kingdom) applied as per the manufacturer's instructions superficially to the lateral aspect of the leg just below the knee. The device stimulates the common peroneal nerve as it passes by the head of fibula, activating the venous muscle pump, and so augmenting venous, arterial, and microvascular flow.^18^ The device delivers a charge-balanced pulse at 1 s intervals, and the settings were adjusted so that a visible twitch of the foot was elicited. Each device nominally lasts 24 h, so each was used for 12 h treatment on two successive days. To maintain skin hygiene, the device is removed and stored overnight between 12-h wear sessions.

*Inclusion Criteria:*

- Aged 18 years or older and able to provide written informed consent.
- Chronic VLU determined to be owing to underlying venous disease following evaluation in a multidisciplinary clinic setting or by a vascular surgeon, general practitioner, or nurse specialist.
- Ulcer size between 3 and 39 cm^2^ at study enrolment.
- Ulcer present for at least 6 weeks but no more than 5 years before study entry.
- Ankle-brachial pressure index (ABPI) of 0.8–1.2 at study entry or within 8 weeks of study entry.
- No clinical infection in the study leg for a minimum of 48 h before study entry.
- No systemic antimicrobial treatment for a minimum of 7 days before study entry prescribed for index ulcer wound infection.

*Exclusion Criteria:*

- Known allergy to any of the protocol-stipulated treatments, or nontolerance of multilayer, multicomponent compression therapy intended for the treatment of VLU.
- History of significant hematological disorders (*e.g.,* sickle cell disease).
- History of deep vein thrombosis (DVT) within 6 months preceding study entry.
- History of pyoderma gangrenosum or other inflammatory ulceration.
- Pregnancy or breast feeding.
- Use of investigational drug or device within 4 weeks before study entry that may interfere with this study.
- Use of any neuromodulation device.
- Surgery during 3 months before study entry (such as abdominal, gynecological, hip or knee replacement)
- Any medication deemed by the investigator to potentially interfere with the study treatment (*e.g.,* systemic steroids).
- Participation in any other clinical study.

**Figure 1.**
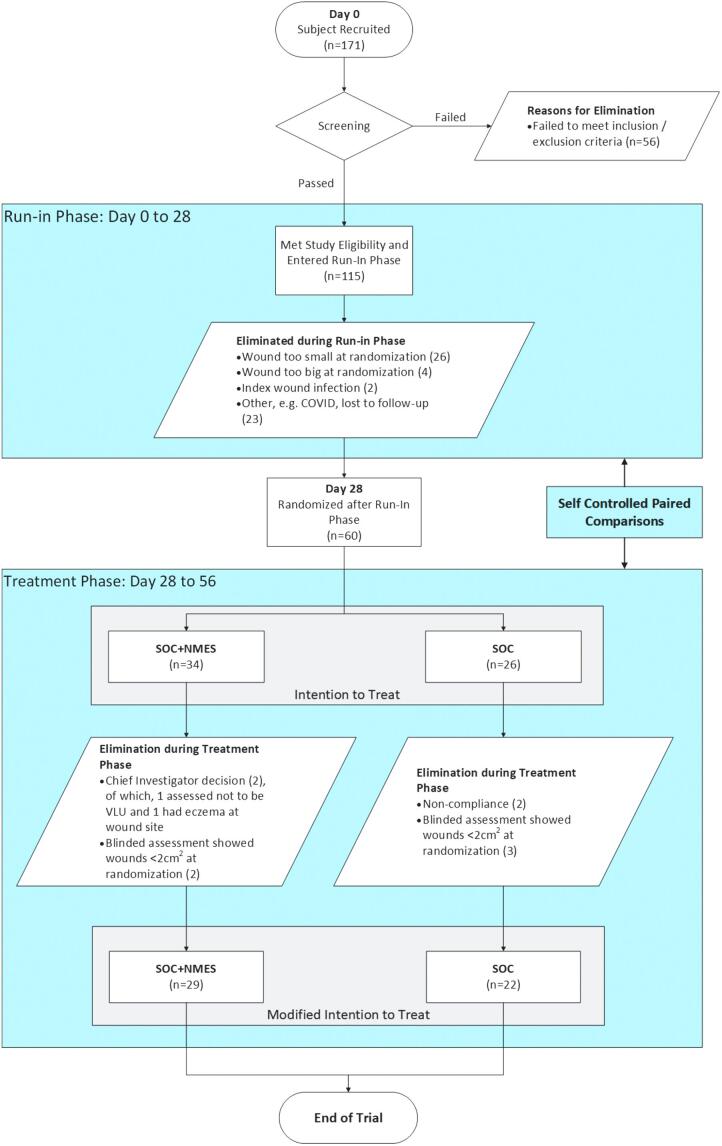
Flow chart illustration of the study design and subject accountability.

A summary graphic of the study design and participant allocation is given in Fig. 1. In the case of both arms, each subject spent 4 weeks on a run-in control phase receiving SOC only. Thereafter, the SOC randomized cohort continued to receive SOC for a further 4 weeks, whereas the NMES randomized cohort received NMES in addition to SOC for a further 4 weeks. The study was not powered to compare healing rates between the two randomized cohorts, but rather for each cohort to compare paired healing rates between intervention phase and run-in phase within the same patient.

At day 0, and at every weekly visit until day 56 (8 weeks), wounds were photographed before debridement using the Aranz SilhouetteStar™ portable digital camera: a noncontact device for imaging and measuring ulcers. All images were sent in random order to an independent wound expert for delineation of the wound perimeter. The assessor was blinded to intervention as well as to the date of each image. All calculations used in the analysis (WMA and PAR) were postprocessed.

The initial wound measurement and four subsequent weekly measurements for each of the two phases of the study (run-in, and treatment) were converted to percentages of the area at the initial visit of the respective phase. For each phase a slope was then calculated by linear regression, to give a PAR per day. Electronic laboratory notebook was not used. Subject data were collected using the Castor EDC platform. The study is reported according to the CONSORT reporting guidelines.^19^

## RESULTS

Table 1 provides the demographics of the subjects, comparing the group randomized to compression only (SOC) with the group randomized to compression plus NMES for 12 h per day (SOC+NMES 12 h). No significant demographic differences were found between groups according to unpaired *t*-tests. Given the immense heterogeneity of venous ulcers in general, it is unsurprising to see some degree of intergroup variation, for example, body mass index (BMI) and age of study ulcer, and this (perhaps inevitable) level of heterogeneity would be problematic in a classic intercohort RCT design. However, in this within patient-controlled design, each subject's intervention phase is compared with his/her own run-in phase, thereby accommodating these differences.

**Table 1.** Subject demographics

	SOC (*n* = 22)		SOC+NMES 12 h Daily (*n* = 29)		*t*-Test
	Mean	SE	Mean	SE	
Age (years)	67.1	2.07	67.8	2.53	0.83
Height (cm)	175.5	2.69	173.58	2.36	0.59
Weight (kg)	84.1	5.98	93.4	4.76	0.23
BMI	27.6	1.89	31.1	1.57	0.16
ABPI	1.1	0.02	1.1	0.02	0.67
Wound size (cm^2^)	10.4	1.22	10.0	1.25	0.81
Age of study VLU at enrolment (days)	477.7	104.20	522.8	89.68	0.74
Age of subject at first VLU (years)	54.0	3.46	59.4	2.95	0.24

ABPI, ankle-brachial pressure index; BMI, body mass index; NMES, neuromuscular electrostimulation; SE, standard error; SOC, standard of care; VLU, venous leg ulcers.

Twenty-six subjects were randomized to SOC and 34 to SOC plus NMES. Of these two groups, in the SOC arm four subjects were excluded: one infection, two wounds too small at randomization, and one inflammation at wound site. For the SOC plus NMES arm: two subjects were excluded owing to nonadherence to NMES therapy (94.1% adherence), and three because of wound being too small at randomization. Overall, 22 subjects completed the SOC arm, and 29 the SOC plus NMES arm (Fig. 1).

Results were analyzed on a Modified Intention To Treat basis. In this instance, a Per Protocol (PP) yields the same dataset, because there were no crossovers between randomized groups.

**Figure 2.**
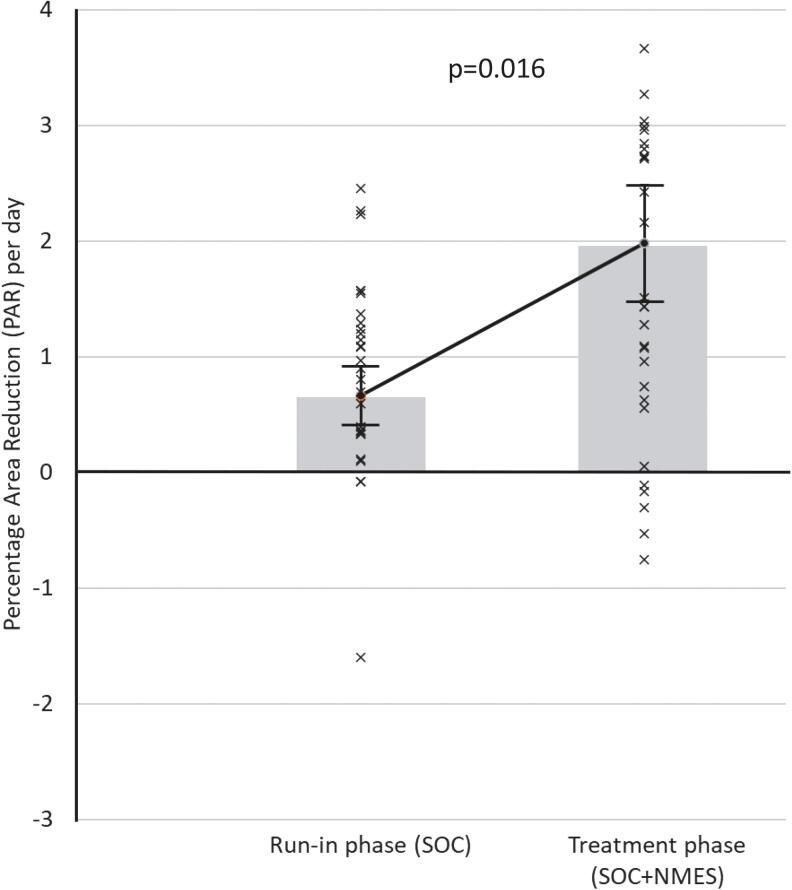
PAR per day for all subjects randomized to the NMES 12 h daily arm (*n* = 29) during 4-week run-in phase (PAR = 0.66% per day: ±0.25% SE) and 4-week treatment phase (PAR = 1.98% per day: ±0.50% SE). NMES, neuromuscular electrostimulation; PAR, percentage area reduction; SE, standard error.

**Figure 3.**
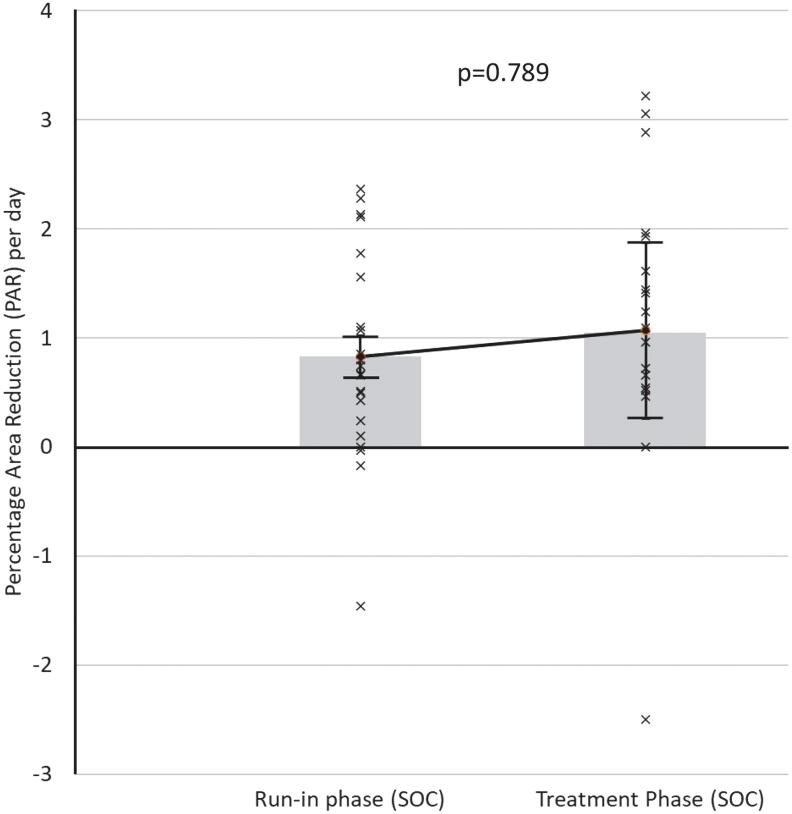
PAR per day for all subjects randomized to the SOC arm (*n* = 22) during 4-week run-in phase (PAR = 0.83% per day: ±0.20% SE) and 4-week treatment phase (PAR = 1.07% per day: ±0.81% SE). SOC, standard of care.

Figure 2 provides the mean healing rates in each group, during run-in phase compared with treatment phase. The treatment phase with NMES as an adjunct to compression showed a wound closure trajectory, over 4 weeks, that was significantly greater (*p* = 0.016) than compression alone in the run-in phase for that cohort. Meanwhile (Fig. 3), no significant difference was found between run-in phase (compression only) and intervention phase (also compression only) in the SOC randomized cohort.

## DISCUSSION

The study was not designed or powered for cohort comparisons between patients randomized to different groups. Whereas within-patient control eliminates between-patient heterogeneity and confounders, these would be reintroduced by any between-group comparisons, so impugning the power of the analysis. Therefore, no comparison is made between the healing rates in the treatment phases of the two groups.

Similarly, any change in healing rate between run-in and treatment phases is made only within groups, not between groups. In this sense, the control group (receiving SOC only throughout run-in and treatment phases) does not serve as a comparator for the NMES group. Rather, it serves as a check that the assumption underpinning the validity of the self-controlled model—a stable baseline healing rate over the period of study—holds true. A healing rate that differed significantly between run-in and treatment phase for the control group would have called this assumption into question.

Of the 171 subjects originally recruited, 120 did not meet the inclusion/exclusion criteria (Fig. 1). This dropout rate is substantial and may have been mitigated by less stringent inclusion/exclusion criteria. However, this would also have increased heterogeneity, and so reduced statistical power. This illustrates the balance to be struck between the internal validity of the study and recruitment efficiency.

Wound healing studies are notoriously difficult to do and rarely demonstrate an effect with statistical significance. It has been noted that they conventionally fail to recruit enough participants.^20^ A Cochrane review^21^ found that even for compression—one of only two wound interventions to meet the threshold of clinical evidence—only 48 RCTs of many thousands of studies in the literature were deemed eligible, of which only 8 had significant stand-alone findings of effectiveness. Studies are frequently improperly designed (*e.g.,* with too few subjects) or imperfectly executed owing to the costs and difficulties in maintaining the requisite high levels of recruitment and assessment over a necessarily long period.^22^ In large part, the difficulties stem from the predominance of complete healing as the outcome of choice, which creates several problems.

Being a binary outcome categorical variable (healed/not healed), it can only be analyzed using frequency statistics, which are the most coarse in terms of statistical significance. This, as well as the immense heterogeneity of wounds, requires cohorts with substantial numbers of subjects to yield statistical power. Given the necessarily lengthy duration of a trial before complete healing is observed in a suitable number of subjects, the logistics of the study become almost prohibitive.

In recent years, experts have called for intermediate endpoints to be recognized.^23,24^ In 2020, the FDA identified five new primary endpoints for consideration: PAR over 4 weeks, reduced infection, reduced pain, increased ambulation, and quality of life.^25^ Of these, PAR is the only truly quantitative metric, and so offers the best opportunity for statistical power in study design.

In the wider world of medical products beyond wounds, there has been a call for the more widespread use of self-controlled studies,^26^ whereby between-patient confounders are inherently eliminated. The self-controlled study may take the form of a contralateral control,^27,28^ or using a separate control site on the patient.^29,30^ This approach is not directly applicable to the study of VLU, as it is rare to find a perfectly matching contralateral ulcer for comparison. One study in pressure ulcers^31^ used a separate part of the same pressure ulcer as a control, but this relies on some assumptions about the homogeneity of the ulcer, and the spatial uniformity of conditions including applied pressure.

A preferable approach may be pre- versus post-treatment self-controlled comparisons, which can be made where within-patient, time-varying confounding factors are absent or small relative to between-patient confounders.^32,33^ This study model is capable of identifying significant differences between treatment regimens with relatively few subjects.^34,35^ A recent review revealed that this model was greatly underused in studies that met the criteria.^36^

To compare healing rate longitudinally using a within-patient control two-stage model before and after the introduction of an intervention, it is first necessary to assume that baseline healing follows a constant linear trajectory over the time interval in question. Only then is it possible or valid to observe a discontinuity in that trajectory when the intervention is introduced. PAR has been shown to follow a linear trajectory with respect to time,^15^ making it a suitable metric for this purpose. Healing rate over 4 weeks has previously been used as the primary outcome in RCTs for comparing interventions.^37^

In this self-controlled study, the addition of intermittent NMES of the common peroneal nerve over a 4-week period doubled the rate of PAR, relative to a 4-week run-in period with compression alone (*p* = 0.016). The device was well tolerated, with 94.1% of subjects adhering to NMES therapy. This level of confidence in the efficacy of the intervention to accelerate wound healing was achieved in 8 weeks, with fewer than 60 subjects. This can be contrasted starkly with the discerning power of a cohort comparison for complete healing.

In the follow-up phase of the published study, 27% of the control group patients had healed by 3 months, whereas 42% of the NMES patients had healed by 3 months. Although apparently substantial, this increase is not statistically significant, owing to the binomial nature of the outcome, and the insufficient number of subjects in the follow-up phase of the study. Assuming healing rates of 27% versus 42%, a sample size calculation for a cohort comparison^38^ yields a required *n* of 412 patients, placing the study in a different category of feasibility even with this substantial effect size.

The methodology presented here is not without limitations: allocation blinding is problematic, because both patient and caregiver can see which intervention is applied; questions exist as to external validity, and the generalizability of findings to a larger population; recruitment, retention, and adherence lead to potential tension between PP and ITT analyses. However, all these limitations plainly also apply to the traditional complete healing RCT model, combined with additional problems resulting from intergroup variances, and the poor discriminant power of a sporadic binary endpoint.

The binomial nature of complete healing as an outcome, although a shortcoming statistically, is also one of the principal reasons for its historical use in wound studies. “Healed/not healed?” has historically been more easily and more reliably observed than “How much healed?,” because it requires no quantification. This landscape is changing rapidly now, as more sophisticated wound measuring technologies emerge, which are ever easier to use, more accurate, less expensive, and more prevalent,^39^ often requiring only a smartphone with a suitable application installed.

## CONCLUSIONS

We need to challenge current dogma that exists in wound studies if we are to make progress in treating patients with chronic wounds. Although it is understandable that complete healing is the desired outcome for such studies to prove efficacy of an intervention, the practical challenges encountered in following this approach are often impossible to overcome. Hence, we still only have minimal proof of any intervention being effective in such wounds. New ways of approaching this problem are required. The design used in this study allows us to calculate a healing rate in an individual using current best practice or SOC. It can be shown that after a 4-week period of observation, patients randomized to continue with the SOC maintained a consistent healing trajectory, whereas the healing rate in the group of patients additionally exposed to NMES improved significantly (*p* = 0.016).

PAR is a useful alternative to complete healing as a metric for comparing the performance of wound healing interventions, and the within-patient controlled trial design allows sensitive discrimination with a relatively small number of subjects over a reasonably short trial period. We suggest that looking at such long-term research design challenges with fresh eyes may prevent many more patients from suffering from chronic wounds for long periods of time when potential interventions are not seen as having proof of efficacy.

## ABOUT THE AUTHORS

**Richard Hillson Bull, FRCP,** is the Consultant Dermatologist at Accelerate CIC. He qualified at Oxford with dermatology training at St. George's and Royal London Hospitals. He has been a dermatology consultant at Barts Health since 1996 with particular interest in leg ulcers, chronic wounds and lymphedema.

**Donna Clements, MSc,** is a Clinical Research Nurse at Norfolk Community Health and Care NHS Trust. She is one of the Senior Research Leaders selected by the National Institute of Healthcare Research to promote an integrated research culture in the UK Health Service.

**Agnes Juguilon Collarte, MA,** is the Tissue Viability Specialist Nurse Lead at the Central London, Hammersmith & Fulham and West London wound clinic Centre for Health & Wellbeing. She has specialized in wound care for 18 years and completed a Masters' degree in “Advancing Practice,” during this time.

**Keith Gordon Harding, FRCS,** is Emeritus Professor at Cardiff University and independent member of Cardiff and Vale University Health Board; he has a distinguished track record in research into wound healing and clinical innovation before recent retirement his clinical practice was exclusively focused on treating patients with complex wound healing problems with a wide range of etiologies.

## Abbreviations and Acronyms

ABPI: ankle-brachial pressure index
BMI: body mass index
DVT: deep vein thrombosis
FDA: Food and Drug Administration
ITT: intention-to-treat
NMES: neuromuscular electrostimulation
PAR: percentage area reduction
PP: per protocol
RCT: randomized controlled trial
SE: standard error
SOC: standard of care
VLU: venous leg ulcer
WMA: wound margin advance
